# Current Rabies Vaccines Do Not Confer Protective Immunity against Divergent Lyssaviruses Circulating in Europe

**DOI:** 10.3390/v11100892

**Published:** 2019-09-24

**Authors:** Juan E. Echevarría, Ashley C. Banyard, Lorraine M. McElhinney, Anthony R. Fooks

**Affiliations:** 1 Instituto de Salud Carlos III, 28220 Madrid, Spain; 2 CIBER de Epidemiología y Salud Pública (CIBERESP), 28029 Madrid, Spain; 3 Department of Virology, Animal and Plant Health Agency (APHA), Addlestone, Surrey KT15 3NB, UK; ashley.banyard@apha.gov.uk (A.C.B.); Lorraine.McElhinney@apha.gov.uk (L.M.M.); Tony.Fooks@apha.gov.uk (A.R.F.); 4 Institute for Infection and Immunity, St. George’s Hospital Medical School, University of London, London SW17 0RE, UK; 5 School of Life Sciences, University of West Sussex, Falmer, West Sussex BN1 9QG, UK; 6 Microbiology and Immunology, Institute of Infection and Global Health, University of Liverpool, Liverpool L69 7BE, UK

**Keywords:** rabies, lyssavirus, vaccine, bat

## Abstract

The use of the rabies vaccine for post-exposure prophylaxis started as early as 1885, revealing a safe and efficient tool to prevent human rabies cases. Preventive vaccination is the basis for the control of canine-mediated rabies, which has already been eliminated from extensive parts of the world, including Europe. Plans to eliminate canine-mediated human rabies by 2030 have been agreed upon by international organisations. However, rabies vaccines are not efficacious against some divergent lyssaviruses. The presence in European indigenous bats of recently described lyssaviruses, which are not neutralised by antibody responses to existing vaccines, as well as the declaration of an imported case of an African lyssavirus, which also escapes vaccine-derived protection, leaves the European health authorities unable to provide efficacious protective vaccines to some potential situations of human exposure. All these circumstances highlight the need for a universal pan-lyssavirus rabies vaccine, able to prevent human rabies in all circumstances.

Rabies is one of the most feared infectious diseases worldwide due to its extreme lethality in animals and humans [1]. For this reason, rabies vaccines for human post-exposure prophylaxis (PEP) were one of the first vaccines developed. The use of the rabies vaccine for PEP started as early as 1885, revealing a safe and efficient tool to prevent human rabies cases. Preventive vaccination is the basis for the control of canine-mediated rabies, which has already been eliminated from extensive parts of the world [1]. However, it is estimated that 59,000 human fatalities are still caused by rabies each year, mostly transmitted by dogs in rural areas of sub-Saharan Africa and south-east Asia. Plans to eliminate canine-mediated human rabies by 2030 have been agreed upon by international organisations [2]. Rabies in dogs and other mammals, specifically from the order *Carnivora*, is caused worldwide by members of the lyssavirus genus, principally rabies virus (RABV), the same virus used as the immunogen in existing human and animal vaccines. Other viruses within this genus are present in bat populations across much of the globe. Interestingly, North and South America are the only continents where RABV is the only lyssavirus present, causing rabies in bats, dogs and other mammals. Elsewhere, across the old world, thirteen different lyssaviruses are known to infect bats and can cause the disease rabies. European bat lyssaviruses type-1 (EBLV-1) and -2 (EBLV-2), Bokeloh bat lyssavirus (BBLV), Aravan lyssavirus (ARAV), Khujand lyssavirus (KHUV), Irkut lyssavirus (IRKV), West Caucasian bat Virus (WCBV), Lleida bat Lyssavirus (LLEBV) and Gannoruwa bat lyssavirus (GBLV) are present in bat populations in Eurasia, Australian bat lyssavirus (ABLV) in Australian bat populations and Duvenhage lyssavirus (DUVV), Lagos Bat lyssavirus (LBV) and Shimoni bat lyssavirus (SHIBV) in African bats [3] (Table 1). 

A further two African viruses, Ikoma lyssavirus (IKOV) and Mokola Virus (MOKV), have been described but have never been detected in bats. Genetic evidence of a putative lyssavirus species detected in Finland, Kotalahti bat lyssavirus, awaits isolation and classification. Lyssaviruses cause neurological disease in mice when infected intracranially under laboratory conditions [4], but only RABV, EBLV-1, EBLV-2, IRKV, ABLV, DUVV and MOKV have been reported in cases of human fatalities [5]. However, human infection with bat lyssavirus has only been reported in regions with the diagnostic capacity to type viruses. It is plausible that cases in Africa and Asia are missed through a lack of laboratory-based human diagnosis or underreporting of rabies generally in endemic areas. Outbreaks of wildlife rabies in terrestrial mammals caused by bat-associated RABV strains have been well documented in the American continent. However, spillover of other bat lyssaviruses to non-bat mammals has been rarely reported [5]. As well as genetic relationships, lyssaviruses are grouped into three different phylogroups, based on both the genetic and antigenic properties of the viruses [6]. The grouping of viruses into phylogroups has important practical consequences for vaccine efficiency, since antigenic cross-reactivity appears restricted to members of the same phylogroup although this warrants further investigation. The majority of lyssaviruses belonging to phylogroup I, including RABV, are neutralised by rabies vaccine-induced antibodies. However, rabies vaccines are not efficacious against LBV, MOKV and SHIBV (phylogroup II), nor against IKOV, WCBV and LLEBV (tentative phylogroup III) [4]. 

All phylogroup I lyssaviruses presently known in Europe are associated with specific bat species belonging to the family *Vespertilionidae*. Cases of EBLV-2 infection have been detected in *Myotis dasycneme* and *Myotis daubentonii,* cases of BBLV infection detected in *Myotis nattereri* [7] and the single identification of the tentative lyssavirus, Kotalahti bat lyssavirus with *Myotis brandtii*. EBLV-1 is associated with more than 95% of the infected bats diagnosed in Europe and, with the exception of sporadic cases, with the serotine bat (*Eptesicus serotinus*), or its sibling species (*Eptesicus isabellinus*) in the southern Iberian Peninsula. Both *Eptesicus* species are synanthropic, which probably drives the higher incidence of EBLV-1 cases detected, since rabies passive surveillance is based on bats submitted by the public and consequently is biased to dead or diseased individuals of the species present near human habitats. In fact, most of the bats submitted for rabies diagnosis are common bats (*Pipistrellus pipistrellus/pygmaeus*), which have been very rarely shown to be infected with lyssaviruses and are not considered rabies reservoirs [7]. Some of the bat species shown to be infected with lyssaviruses in the Asian part of the continent also inhabit Europe and consequently their corresponding lyssaviruses are likely to be present. Such pathogens include ARAV (*Myotis blythii*) and KHUV (*Myotis mystacinus*). Rabies vaccine-induced antibodies neutralise all these viruses and consequently PEP is believed to be efficacious for the prevention of human infection by these viruses [8]. 

In 2003, a new lyssavirus, WCBV, was detected in a cave bat (*Miniopterus schreibersii*) captured in the northern region of the Caucasus. More recently, another lyssavirus, LLEBV, was reported in the same bat species in the city of Lleida, at the north-east of the Iberian Peninsula in Spain [3]. An additional case from France in the same bat species has been recently communicated [9] (Figure 1). 

Both WCBV and LLEBV are amongst the most phylogenetically distant to phylogroup I and are not neutralised by vaccine-induced antibodies in vitro. Moreover, currently available vaccines have been shown not to be efficient for preventing infection by these two viruses during challenge experiments in a mouse model [4,10]. Fortunately, there have not been any reports of human exposure to these lyssaviruses.

In 1999, a fruit bat (*Rousettus aegyptiacus*) imported from Africa into Belgium and then transferred to the city of Bourdeaux in France was shown to be infected with LBV (Table 1). LBV is an African lyssavirus for which rabies vaccines do not confer protective immunity [3]. *Rousettus aegyptiacus* breed readily in captivity and are widespread in zoos around the world and even kept as pets by the general public. This species is the only fruit bat occurring in Europe, becoming naturalised in the Canary Islands (Spain) and also on the island of Cyprus (Eastern Mediterranean). Interestingly, the barrier effect of the Strait of Gibraltar on bat populations is different depending on the species considered and connections between African and European populations have been demonstrated in some cases, including some that are known to be reservoirs of lyssaviruses, as in the case of *E. isabellinus* [11]. This migration opens the possibility of lyssavirus interchange between Africa and Europe, which eventually could include unknown divergent viruses, since the knowledge of lyssavirus diversity in northern Africa has not been sufficiently investigated.

Bat rabies has been considered a threat that can be safely managed in Europe with the combination of good surveillance and immediate PEP. However, the presence in European indigenous bats of recently described lyssaviruses, which are not neutralised by antibody responses to existing vaccines, as well as the declaration of an imported case of an African lyssavirus which also escapes vaccine-derived protection, leaves the European health authorities unable to provide efficacious protective vaccines to some potential situations of human exposure to phylogroups II and III bat lyssaviruses. It is true that captive *Rousettus aegyptiacus* are bred locally in Europe and *Miniopterus schreibersii* is not usually linked to urban or suburban environments and rarely interacts with humans. In fact, both bat species are underrepresented in the series of passive surveillance in Europe. However, although low, the risk of human exposure to their associated lyssaviruses exists. This risk must be considered in the global plan to eliminate human rabies, especially should the elimination of dog-mediated human rabies by the 2030 target be successful. New lyssaviruses are discovered with increased frequency. Moreover, there are several studies reporting lyssavirus antibodies on a wide spectrum of bat species in Europe [7], suggesting that, either the epidemiology of the already known European lyssaviruses is more complex than the passive surveillance shows, or there are other presently unknown lyssavirus species cross-reactive on antibody tests, for which the efficiency of the presently available vaccines is not known. All these circumstances highlight the need for a universal pan-lyssavirus rabies vaccine, able to prevent human rabies in all circumstances [5]. Technology is available to develop and validate a broad-spectrum lyssavirus vaccine [5]. The requirement to have to extend the application of any broad-range vaccine will depend on an increase in risk post dog-mediated human rabies elimination.

## Figures and Tables

**Figure 1:**
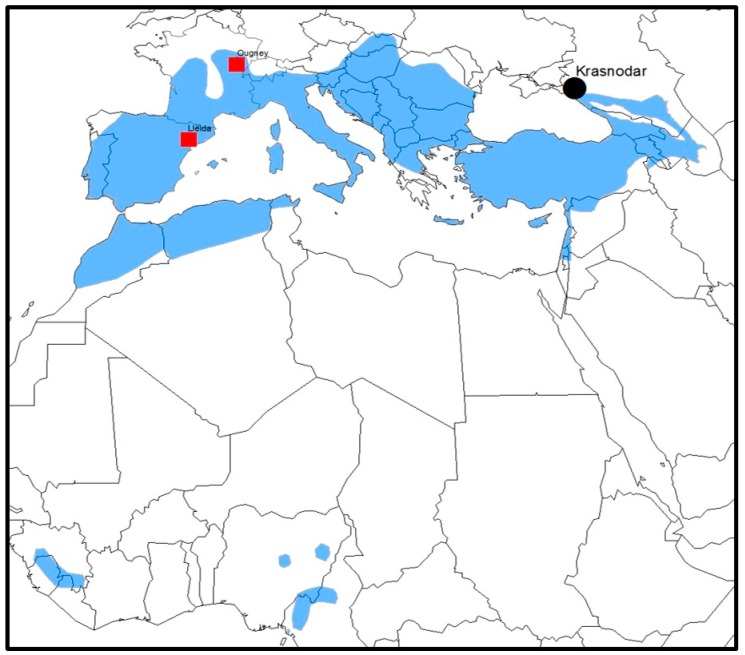
Location of Lleida Bat lyssavirus (LLEBV) cases (red squares) and West Caucasian Bat lyssavirus (WCBV) (black circle). Geographical distribution of *Miniopterus schreibersii* (blue shadow). Host range data derived from IUCN (www.IUCN.org).

**Table 1:** Bat lyssaviruses.

Lyssavirus Species	Phylogroup	Bat Species Most Often Associated with Lyssavirus Infection	Human Cases	Countries Reporting Lyssavirus in Bats
Aravan lyssavirus (ARAV)	I	*Myotis blythi*	No	Kyrgyzstan
Australian bat lyssavirus (ABLV)	I	*Pteropus alecto* *Pteropus scapulatus* *Pteropus poliocephalus* *Pteropus conspicillatus* *Saccolaimus flaviventris*	Yes, three	Australia
Bokeloh bat lyssavirus (BBLV)	I	*Myotis nattereri*	No	Germany, France, Poland
Duvenhage lyssavirus (DUVV)	I	*Miniopterus sp* *Nycteris thebaica*	Yes, three	South Africa, Kenya Zimbabwe
European bat 1 Lyssavirus (EBLV-1)	I	*Eptesicus serotinus* *Eptesicus isabellinus* *Vespertilio murinus* *Pipistrellus nathusii* *Pipistrellus pipistrellus*	Yes, two	France, Germany, The Netherlands, Poland, Denmark, Spain, Ukraine, Russia, Hungary
European bat 2 lyssavirus (EBLV-2)	I	*Myotis daubentonii* *Myotis dasycneme*	Yes, two	The Netherlands, Switzerland, United Kingdom, Germany, Finland, Norway, Denmark
Gannoruwa bat lyssavirus (GBLV)	I	*Pteropus medius*	No	Sri Lanka
Irkut lyssavirus (IRKV)	I	*Murina leucogaster*	Yes, one	Russian Federation, China
Kotolahti Bat Lyssavirus (KBLV)$	I	*Myotis brandtii*	No	Finland
Khujand lyssavirus (KHUV)	I	*Myotis mystacinus*	No	Tajikistan
Lagos bat lyssavirus (LBV)	II	*Eidolon helvum* *Rousettus aegyptiacus* *Micropteropus pussilus* *Nycteris gambiensis* *Epomophorus wahlbergi*	No	Nigeria, Senegal, Ghana, Guinea, Kenya, France (ex-Togo or Egypt), Central African Republic, South Africa
Lleida bat lyssavirus (LLEBV)	III	*Miniopterus schreibersii*	No	Spain, France
Rabies lyssavirus (RABV)	I	Multiple	Yes, 59,000/year. Mostly transmitted by dogs	North and South America
Shimoni bat lyssavirus (SHIBV)	II	*Hipposideros commersoni*	No	Kenya
Taiwan bat lyssavirus (TWBLV)$	I	*Pipistrellus abramus*	No	Taiwan
West Caucasian bat lyssavirus (WCBV)	III	*Miniopterus schreibersii*	No	Russian Federation

$ Not currently classified within the lyssavirus genus.
